# Supplementary material for: Association of polycyclic aromatic hydrocarbons in moss with blood biomarker among nearby residents in Portland, Oregon
Source: PLoS One. 2022 Dec 19;17(12):e0279207. doi: 10.1371/journal.pone.0279207 (PMC9762581; doi:10.1371/journal.pone.0279207)

# Table 1: Descriptive statistics of raw PAH measurements; non-detects not imputed, compelte case analysis

## The MEANS Procedure

| Variable | N | N Miss | Mean | Std Dev | Median | Minimum | Maximum |
|---|---|---|---|---|---|---|---|
| adducts | 32 | 21 | 11.8343750 | 3.1862213 | 11.5000000 | 7.9000000 | 22.9000000 |
| _1_Methylnaphthalene | 47 | 6 | 5.9510638 | 8.3488058 | 3.8800000 | 1.4800000 | 46.9000000 |
| _2_Methylnaphthalene | 53 | 0 | 6.5877358 | 9.4918510 | 5.2500000 | 2.3200000 | 72.4000000 |
| Acenaphthene | 28 | 25 | 4.0982143 | 4.0138937 | 3.2300000 | 1.7700000 | 23.5000000 |
| Acenaphthylene | 25 | 28 | 4.1456000 | 4.1753376 | 3.0000000 | 1.7000000 | 23.0000000 |
| Anthracene | 49 | 4 | 3.8744898 | 1.5030387 | 3.7100000 | 1.6700000 | 10.3000000 |
| Benzo_a_anthracene | 53 | 0 | 20.0189623 | 17.0227661 | 16.3000000 | 6.2900000 | 124.0000000 |
| Benzo_a_pyrene | 42 | 11 | 15.0242857 | 6.7326265 | 15.0500000 | 3.8200000 | 31.3000000 |
| Benzo_b_fluoranthene | 53 | 0 | 22.5716981 | 10.7204544 | 21.3000000 | 3.3300000 | 66.5000000 |
| Benzo_ghi_perylene | 49 | 4 | 22.6674490 | 9.7949063 | 21.1000000 | 7.6250000 | 68.5000000 |
| Benzo_k_fluoranthene | 52 | 1 | 15.8488462 | 5.2618049 | 15.3000000 | 6.5700000 | 29.6000000 |
| Chrysene | 53 | 0 | 38.5783019 | 12.8428351 | 36.1000000 | 14.2000000 | 65.6000000 |
| Dibenzo_a_h_anthracene | 14 | 39 | 10.4217857 | 4.8389094 | 9.1250000 | 4.5250000 | 20.6000000 |
| Dibenzofuran | 48 | 5 | 5.9760417 | 6.9946112 | 4.5200000 | 2.3700000 | 41.8000000 |
| Fluoranthene | 53 | 0 | 34.4339623 | 11.1330406 | 32.7000000 | 13.4000000 | 66.7000000 |
| Fluorene | 19 | 34 | 4.8000000 | 5.3415260 | 2.9000000 | 1.8000000 | 20.5000000 |
| Ieno_1_2_3_cd_pyrene | 35 | 18 | 10.9688571 | 4.7298189 | 9.8600000 | 3.2300000 | 25.7000000 |
| Naphthalene | 53 | 0 | 13.1359434 | 16.3082079 | 9.8400000 | 3.8900000 | 97.7000000 |
| Perylene | 49 | 4 | 16.5740816 | 6.2898761 | 16.2000000 | 2.4300000 | 39.5000000 |
| Phenanthrene | 53 | 0 | 20.8877358 | 5.7459091 | 21.4000000 | 10.1000000 | 35.6000000 |
| Pyrene | 53 | 0 | 28.5150943 | 10.0684226 | 29.1000000 | 10.7500000 | 68.4000000 |
| age | 53 | 0 | 50.5283019 | 9.0076971 | 52.0000000 | 30.0000000 | 68.0000000 |

# Table 2: Correlation (rank) of PAH and adducts: complete case analysis

## The CORR Procedure

| | |
|---|---|
| **21 Variables:** | adducts _1_Methylnaphthalene _2_Methylnaphthalene Acenaphthene Acenaphthylene Anthracene Benzo_a_anthracene Benzo_a_pyrene Benzo_b_fluoranthene Benzo_ghi_perylene Benzo_k_fluoranthene Chrysene Dibenzo_a_h_anthracene Dibenzofuran Fluoranthene Fluorene Ieno_1_2_3_cd_pyrene Naphthalene Perylene Phenanthrene Pyrene |

| Simple Statistics | | | | | | |
|---|---|---|---|---|---|---|
| **Variable** | **N** | **Mean** | **Std Dev** | **Median** | **Minimum** | **Maximum** |
| **adducts** | 32 | 11.83438 | 3.18622 | 11.50000 | 7.90000 | 22.90000 |
| **_1_Methylnaphthalene** | 47 | 5.95106 | 8.34881 | 3.88000 | 1.48000 | 46.90000 |
| **_2_Methylnaphthalene** | 53 | 6.58774 | 9.49185 | 5.25000 | 2.32000 | 72.40000 |
| **Acenaphthene** | 28 | 4.09821 | 4.01389 | 3.23000 | 1.77000 | 23.50000 |
| **Acenaphthylene** | 25 | 4.14560 | 4.17534 | 3.00000 | 1.70000 | 23.00000 |
| **Anthracene** | 49 | 3.87449 | 1.50304 | 3.71000 | 1.67000 | 10.30000 |
| **Benzo_a_anthracene** | 53 | 20.01896 | 17.02277 | 16.30000 | 6.29000 | 124.00000 |
| **Benzo_a_pyrene** | 42 | 15.02429 | 6.73263 | 15.05000 | 3.82000 | 31.30000 |
| **Benzo_b_fluoranthene** | 53 | 22.57170 | 10.72045 | 21.30000 | 3.33000 | 66.50000 |
| **Benzo_ghi_perylene** | 49 | 22.66745 | 9.79491 | 21.10000 | 7.62500 | 68.50000 |
| **Benzo_k_fluoranthene** | 52 | 15.84885 | 5.26180 | 15.30000 | 6.57000 | 29.60000 |
| **Chrysene** | 53 | 38.57830 | 12.84284 | 36.10000 | 14.20000 | 65.60000 |
| **Dibenzo_a_h_anthracene** | 14 | 10.42179 | 4.83891 | 9.12500 | 4.52500 | 20.60000 |
| **Dibenzofuran** | 48 | 5.97604 | 6.99461 | 4.52000 | 2.37000 | 41.80000 |
| **Fluoranthene** | 53 | 34.43396 | 11.13304 | 32.70000 | 13.40000 | 66.70000 |
| **Fluorene** | 19 | 4.80000 | 5.34153 | 2.90000 | 1.80000 | 20.50000 |
| **Ieno_1_2_3_cd_pyrene** | 35 | 10.96886 | 4.72982 | 9.86000 | 3.23000 | 25.70000 |
| **Naphthalene** | 53 | 13.13594 | 16.30821 | 9.84000 | 3.89000 | 97.70000 |
| **Perylene** | 49 | 16.57408 | 6.28988 | 16.20000 | 2.43000 | 39.50000 |
| **Phenanthrene** | 53 | 20.88774 | 5.74591 | 21.40000 | 10.10000 | 35.60000 |
| **Pyrene** | 53 | 28.51509 | 10.06842 | 29.10000 | 10.75000 | 68.40000 |

# Table 2: Correlation (rank) of PAH and adducts: complete case analysis

## The CORR Procedure

| Spearman Correlation Coefficients<br>Prob > \|r\| under H0: Rho=0<br>Number of Observations | | | | | | |
|---|---|---|---|---|---|---|
| | **adducts** | **_1_Methylnaphthalene** | **_2_Methylnaphthalene** | **Acenaphthene** | **Acenaphthylene** | **Anthracene** |
| **adducts** | 1.00000<br><br>32 | 0.20694<br>0.3004<br>27 | -0.00303<br>0.9869<br>32 | -0.03498<br>0.9015<br>15 | -0.23468<br>0.4628<br>12 | 0.05029<br>0.7994<br>28 |
| **_1_Methylnaphthalene** | 0.20694<br>0.3004<br>27 | 1.00000<br><br>47 | 0.73012<br><.0001<br>47 | 0.36133<br>0.0641<br>27 | 0.40500<br>0.0446<br>25 | 0.43318<br>0.0030<br>45 |
| **_2_Methylnaphthalene** | -0.00303<br>0.9869<br>32 | 0.73012<br><.0001<br>47 | 1.00000<br><br>53 | 0.43516<br>0.0206<br>28 | 0.35380<br>0.0827<br>25 | 0.46582<br>0.0007<br>49 |
| **Acenaphthene** | -0.03498<br>0.9015<br>15 | 0.36133<br>0.0641<br>27 | 0.43516<br>0.0206<br>28 | 1.00000<br><br>28 | 0.71779<br>0.0012<br>17 | 0.31298<br>0.1195<br>26 |
| **Acenaphthylene** | -0.23468<br>0.4628<br>12 | 0.40500<br>0.0446<br>25 | 0.35380<br>0.0827<br>25 | 0.71779<br>0.0012<br>17 | 1.00000<br><br>25 | 0.33689<br>0.1074<br>24 |
| **Anthracene** | 0.05029<br>0.7994<br>28 | 0.43318<br>0.0030<br>45 | 0.46582<br>0.0007<br>49 | 0.31298<br>0.1195<br>26 | 0.33689<br>0.1074<br>24 | 1.00000<br><br>49 |
| **Benzo_a_anthracene** | 0.16375<br>0.3705<br>32 | 0.30991<br>0.0340<br>47 | 0.41444<br>0.0020<br>53 | -0.02382<br>0.9042<br>28 | -0.19827<br>0.3421<br>25 | 0.49055<br>0.0003<br>49 |
| **Benzo_a_pyrene** | 0.03311<br>0.8752<br>25 | 0.51728<br>0.0009<br>38 | 0.52817<br>0.0003<br>42 | 0.18560<br>0.3965<br>23 | 0.25934<br>0.2563<br>21 | 0.56484<br>0.0002<br>38 |
| **Benzo_b_fluoranthene** | 0.05899<br>0.7485<br>32 | 0.07751<br>0.6046<br>47 | 0.37952<br>0.0051<br>53 | -0.00616<br>0.9752<br>28 | -0.12303<br>0.5580<br>25 | 0.41774<br>0.0028<br>49 |
| **Benzo_ghi_perylene** | 0.15001<br>0.4373<br>29 | 0.30029<br>0.0504<br>43 | 0.50234<br>0.0002<br>49 | 0.16732<br>0.4139<br>26 | 0.17067<br>0.4362<br>23 | 0.72295<br><.0001<br>45 |
| **Benzo_k_fluoranthene** | 0.06877<br>0.7132<br>31 | 0.08601<br>0.5698<br>46 | 0.31442<br>0.0232<br>52 | 0.31042<br>0.1151<br>27 | -0.08879<br>0.6799<br>24 | 0.43314<br>0.0021<br>48 |
| **Chrysene** | 0.15430<br>0.3991<br>32 | 0.36643<br>0.0113<br>47 | 0.41358<br>0.0021<br>53 | -0.12995<br>0.5099<br>28 | -0.28456<br>0.1680<br>25 | 0.40108<br>0.0043<br>49 |
| **Dibenzo_a_h_anthracene** | 0.15569<br>0.7128<br>8 | 0.18785<br>0.5389<br>13 | 0.22737<br>0.4344<br>14 | 0.42169<br>0.2981<br>8 | 0.28834<br>0.4191<br>10 | 0.41989<br>0.1532<br>13 |
| **Dibenzofuran** | -0.09722<br>0.6226<br>28 | 0.23695<br>0.1171<br>45 | 0.29630<br>0.0409<br>48 | 0.43072<br>0.0280<br>26 | 0.15681<br>0.4749<br>23 | 0.26648<br>0.0768<br>45 |
| **Fluoranthene** | 0.17897<br>0.3270<br>32 | 0.17098<br>0.2505<br>47 | 0.44561<br>0.0008<br>53 | -0.04929<br>0.8033<br>28 | -0.08664<br>0.6805<br>25 | 0.59100<br><.0001<br>49 |
| **Fluorene** | 0.17576<br>0.6272<br>10 | 0.51775<br>0.0278<br>18 | 0.64570<br>0.0028<br>19 | 0.32773<br>0.3893<br>9 | 0.84404<br>0.0021<br>10 | 0.33392<br>0.1623<br>19 |

# Table 2: Correlation (rank) of PAH and adducts: complete case analysis

## The CORR Procedure

| Spearman Correlation Coefficients<br>Prob > \|r\| under H0: Rho=0<br>Number of Observations | | | | |
|---|---|---|---|---|
| | **Benzo_a_anthracene** | **Benzo_a_pyrene** | **Benzo_b_fluoranthene** | **Benzo_ghi_perylene** |
| **adducts** | 0.16375<br>0.3705<br>32 | 0.03311<br>0.8752<br>25 | 0.05899<br>0.7485<br>32 | 0.15001<br>0.4373<br>29 |
| **_1_Methylnaphthalene** | 0.30991<br>0.0340<br>47 | 0.51728<br>0.0009<br>38 | 0.07751<br>0.6046<br>47 | 0.30029<br>0.0504<br>43 |
| **_2_Methylnaphthalene** | 0.41444<br>0.0020<br>53 | 0.52817<br>0.0003<br>42 | 0.37952<br>0.0051<br>53 | 0.50234<br>0.0002<br>49 |
| **Acenaphthene** | -0.02382<br>0.9042<br>28 | 0.18560<br>0.3965<br>23 | -0.00616<br>0.9752<br>28 | 0.16732<br>0.4139<br>26 |
| **Acenaphthylene** | -0.19827<br>0.3421<br>25 | 0.25934<br>0.2563<br>21 | -0.12303<br>0.5580<br>25 | 0.17067<br>0.4362<br>23 |
| **Anthracene** | 0.49055<br>0.0003<br>49 | 0.56484<br>0.0002<br>38 | 0.41774<br>0.0028<br>49 | 0.72295<br><.0001<br>45 |
| **Benzo_a_anthracene** | 1.00000<br><br>53 | 0.74246<br><.0001<br>42 | 0.47207<br>0.0004<br>53 | 0.52558<br>0.0001<br>49 |
| **Benzo_a_pyrene** | 0.74246<br><.0001<br>42 | 1.00000<br><br>42 | 0.53344<br>0.0003<br>42 | 0.71216<br><.0001<br>41 |
| **Benzo_b_fluoranthene** | 0.47207<br>0.0004<br>53 | 0.53344<br>0.0003<br>42 | 1.00000<br><br>53 | 0.61715<br><.0001<br>49 |
| **Benzo_ghi_perylene** | 0.52558<br>0.0001<br>49 | 0.71216<br><.0001<br>41 | 0.61715<br><.0001<br>49 | 1.00000<br><br>49 |
| **Benzo_k_fluoranthene** | 0.39311<br>0.0039<br>52 | 0.51127<br>0.0005<br>42 | 0.56157<br><.0001<br>52 | 0.54625<br><.0001<br>49 |
| **Chrysene** | 0.90054<br><.0001<br>53 | 0.74987<br><.0001<br>42 | 0.43937<br>0.0010<br>53 | 0.42714<br>0.0022<br>49 |
| **Dibenzo_a_h_anthracene** | 0.38631<br>0.1725<br>14 | 0.12195<br>0.7372<br>10 | 0.30684<br>0.2859<br>14 | 0.19647<br>0.5008<br>14 |
| **Dibenzofuran** | -0.01477<br>0.9206<br>48 | -0.02553<br>0.8791<br>38 | 0.09744<br>0.5100<br>48 | 0.03456<br>0.8237<br>44 |
| **Fluoranthene** | 0.66944<br><.0001<br>53 | 0.73086<br><.0001<br>42 | 0.75038<br><.0001<br>53 | 0.64861<br><.0001<br>49 |
| **Fluorene** | -0.10009<br>0.6835<br>19 | 0.07307<br>0.7732<br>18 | -0.05403<br>0.8261<br>19 | 0.16493<br>0.5131<br>18 |

# Table 2: Correlation (rank) of PAH and adducts: complete case analysis

## The CORR Procedure

**Spearman Correlation Coefficients**
**Prob > |r| under H0: Rho=0**
**Number of Observations**

| | Benzo_k_fluoranthene | Chrysene | Dibenzo_a_h_anthracene | Dibenzofuran | Fluoranthene | Fluorene |
|---|---|---|---|---|---|---|
| **adducts** | 0.06877<br>0.7132<br>31 | 0.15430<br>0.3991<br>32 | 0.15569<br>0.7128<br>8 | -0.09722<br>0.6226<br>28 | 0.17897<br>0.3270<br>32 | 0.17576<br>0.6272<br>10 |
| **_1_Methylnaphthalene** | 0.08601<br>0.5698<br>46 | 0.36643<br>0.0113<br>47 | 0.18785<br>0.5389<br>13 | 0.23695<br>0.1171<br>45 | 0.17098<br>0.2505<br>47 | 0.51775<br>0.0278<br>18 |
| **_2_Methylnaphthalene** | 0.31442<br>0.0232<br>52 | 0.41358<br>0.0021<br>53 | 0.22737<br>0.4344<br>14 | 0.29630<br>0.0409<br>48 | 0.44561<br>0.0008<br>53 | 0.64570<br>0.0028<br>19 |
| **Acenaphthene** | 0.31042<br>0.1151<br>27 | -0.12995<br>0.5099<br>28 | 0.42169<br>0.2981<br>8 | 0.43072<br>0.0280<br>26 | -0.04929<br>0.8033<br>28 | 0.32773<br>0.3893<br>9 |
| **Acenaphthylene** | -0.08879<br>0.6799<br>24 | -0.28456<br>0.1680<br>25 | 0.28834<br>0.4191<br>10 | 0.15681<br>0.4749<br>23 | -0.08664<br>0.6805<br>25 | 0.84404<br>0.0021<br>10 |
| **Anthracene** | 0.43314<br>0.0021<br>48 | 0.40108<br>0.0043<br>49 | 0.41989<br>0.1532<br>13 | 0.26648<br>0.0768<br>45 | 0.59100<br><.0001<br>49 | 0.33392<br>0.1623<br>19 |
| **Benzo_a_anthracene** | 0.39311<br>0.0039<br>52 | 0.90054<br><.0001<br>53 | 0.38631<br>0.1725<br>14 | -0.01477<br>0.9206<br>48 | 0.66944<br><.0001<br>53 | -0.10009<br>0.6835<br>19 |
| **Benzo_a_pyrene** | 0.51127<br>0.0005<br>42 | 0.74987<br><.0001<br>42 | 0.12195<br>0.7372<br>10 | -0.02553<br>0.8791<br>38 | 0.73086<br><.0001<br>42 | 0.07307<br>0.7732<br>18 |
| **Benzo_b_fluoranthene** | 0.56157<br><.0001<br>52 | 0.43937<br>0.0010<br>53 | 0.30684<br>0.2859<br>14 | 0.09744<br>0.5100<br>48 | 0.75038<br><.0001<br>53 | -0.05403<br>0.8261<br>19 |
| **Benzo_ghi_perylene** | 0.54625<br><.0001<br>49 | 0.42714<br>0.0022<br>49 | 0.19647<br>0.5008<br>14 | 0.03456<br>0.8237<br>44 | 0.64861<br><.0001<br>49 | 0.16493<br>0.5131<br>18 |
| **Benzo_k_fluoranthene** | 1.00000<br><br>52 | 0.51866<br><.0001<br>52 | 0.46409<br>0.0946<br>14 | 0.24007<br>0.1041<br>47 | 0.67789<br><.0001<br>52 | -0.04004<br>0.8707<br>19 |
| **Chrysene** | 0.51866<br><.0001<br>52 | 1.00000<br><br>53 | 0.36022<br>0.2058<br>14 | 0.09087<br>0.5391<br>48 | 0.71833<br><.0001<br>53 | -0.07529<br>0.7594<br>19 |
| **Dibenzo_a_h_anthracene** | 0.46409<br>0.0946<br>14 | 0.36022<br>0.2058<br>14 | 1.00000<br><br>14 | 0.13380<br>0.6785<br>12 | 0.47020<br>0.0898<br>14 | -0.33333<br>0.6667<br>4 |
| **Dibenzofuran** | 0.24007<br>0.1041<br>47 | 0.09087<br>0.5391<br>48 | 0.13380<br>0.6785<br>12 | 1.00000<br><br>48 | 0.16745<br>0.2553<br>48 | 0.59499<br>0.0092<br>18 |
| **Fluoranthene** | 0.67789<br><.0001<br>52 | 0.71833<br><.0001<br>53 | 0.47020<br>0.0898<br>14 | 0.16745<br>0.2553<br>48 | 1.00000<br><br>53 | 0.05049<br>0.8374<br>19 |
| **Fluorene** | -0.04004<br>0.8707<br>19 | -0.07529<br>0.7594<br>19 | -0.33333<br>0.6667<br>4 | 0.59499<br>0.0092<br>18 | 0.05049<br>0.8374<br>19 | 1.00000<br><br>19 |

# Table 2: Correlation (rank) of PAH and adducts: complete case analysis

## The CORR Procedure

Spearman Correlation Coefficients
Prob > |r| under H0: Rho=0
Number of Observations

| | Ieno_1_2_3_cd_pyrene | Naphthalene | Perylene | Phenanthrene | Pyrene |
|---|---|---|---|---|---|
| **adducts** | 0.38926<br>0.1103<br>18 | 0.33915<br>0.0576<br>32 | 0.16385<br>0.3957<br>29 | 0.01597<br>0.9309<br>32 | 0.17193<br>0.3468<br>32 |
| **_1_Methylnaphthalene** | 0.46913<br>0.0078<br>31 | 0.56500<br><.0001<br>47 | 0.37368<br>0.0136<br>43 | 0.26326<br>0.0738<br>47 | 0.08797<br>0.5566<br>47 |
| **_2_Methylnaphthalene** | 0.41866<br>0.0123<br>35 | 0.37374<br>0.0058<br>53 | 0.55513<br><.0001<br>49 | 0.47422<br>0.0003<br>53 | 0.42260<br>0.0016<br>53 |
| **Acenaphthene** | 0.26680<br>0.2845<br>18 | 0.16512<br>0.4011<br>28 | 0.30645<br>0.1362<br>25 | 0.21194<br>0.2789<br>28 | -0.00233<br>0.9906<br>28 |
| **Acenaphthylene** | 0.30958<br>0.2266<br>17 | 0.20697<br>0.3209<br>25 | 0.46523<br>0.0291<br>22 | 0.32878<br>0.1086<br>25 | -0.05721<br>0.7859<br>25 |
| **Anthracene** | 0.57017<br>0.0007<br>32 | 0.42870<br>0.0021<br>49 | 0.75429<br><.0001<br>45 | 0.68045<br><.0001<br>49 | 0.53600<br><.0001<br>49 |
| **Benzo_a_anthracene** | 0.47840<br>0.0037<br>35 | 0.39508<br>0.0034<br>53 | 0.59144<br><.0001<br>49 | 0.48846<br>0.0002<br>53 | 0.56389<br><.0001<br>53 |
| **Benzo_a_pyrene** | 0.62087<br>0.0003<br>30 | 0.44551<br>0.0031<br>42 | 0.69353<br><.0001<br>40 | 0.50308<br>0.0007<br>42 | 0.61207<br><.0001<br>42 |
| **Benzo_b_fluoranthene** | 0.57383<br>0.0003<br>35 | -0.02895<br>0.8370<br>53 | 0.57603<br><.0001<br>49 | 0.55138<br><.0001<br>53 | 0.74884<br><.0001<br>53 |
| **Benzo_ghi_perylene** | 0.72480<br><.0001<br>35 | 0.41045<br>0.0034<br>49 | 0.74916<br><.0001<br>48 | 0.63280<br><.0001<br>49 | 0.69251<br><.0001<br>49 |
| **Benzo_k_fluoranthene** | 0.43182<br>0.0096<br>35 | 0.08218<br>0.5625<br>52 | 0.69012<br><.0001<br>49 | 0.44329<br>0.0010<br>52 | 0.68057<br><.0001<br>52 |
| **Chrysene** | 0.43185<br>0.0096<br>35 | 0.45035<br>0.0007<br>53 | 0.60201<br><.0001<br>49 | 0.47696<br>0.0003<br>53 | 0.56698<br><.0001<br>53 |
| **Dibenzo_a_h_anthracene** | 0.06077<br>0.8437<br>13 | 0.20088<br>0.4911<br>14 | 0.20971<br>0.4718<br>14 | 0.02428<br>0.9343<br>14 | 0.23179<br>0.4252<br>14 |
| **Dibenzofuran** | -0.00955<br>0.9586<br>32 | 0.17628<br>0.2307<br>48 | 0.18727<br>0.2235<br>44 | 0.36358<br>0.0111<br>48 | 0.19856<br>0.1761<br>48 |
| **Fluoranthene** | 0.55391<br>0.0006<br>35 | 0.26966<br>0.0509<br>53 | 0.76430<br><.0001<br>49 | 0.73455<br><.0001<br>53 | 0.93139<br><.0001<br>53 |
| **Fluorene** | 0.07658<br>0.7947<br>14 | 0.27559<br>0.2535<br>19 | 0.02609<br>0.9208<br>17 | 0.30912<br>0.1978<br>19 | 0.17102<br>0.4839<br>19 |

# Table 2: Correlation (rank) of PAH and adducts: complete case analysis

## The CORR Procedure

**Spearman Correlation Coefficients**
**Prob > |r| under H0: Rho=0**
**Number of Observations**

| | adducts | _1_Methylnaphthalene | _2_Methylnaphthalene | Acenaphthene | Acenaphthylene | Anthracene |
|---|---|---|---|---|---|---|
| **Ieno_1_2_3_cd_pyrene** | 0.38926<br>0.1103<br>18 | 0.46913<br>0.0078<br>31 | 0.41866<br>0.0123<br>35 | 0.26680<br>0.2845<br>18 | 0.30958<br>0.2266<br>17 | 0.57017<br>0.0007<br>32 |
| **Naphthalene** | 0.33915<br>0.0576<br>32 | 0.56500<br><.0001<br>47 | 0.37374<br>0.0058<br>53 | 0.16512<br>0.4011<br>28 | 0.20697<br>0.3209<br>25 | 0.42870<br>0.0021<br>49 |
| **Perylene** | 0.16385<br>0.3957<br>29 | 0.37368<br>0.0136<br>43 | 0.55513<br><.0001<br>49 | 0.30645<br>0.1362<br>25 | 0.46523<br>0.0291<br>22 | 0.75429<br><.0001<br>45 |
| **Phenanthrene** | 0.01597<br>0.9309<br>32 | 0.26326<br>0.0738<br>47 | 0.47422<br>0.0003<br>53 | 0.21194<br>0.2789<br>28 | 0.32878<br>0.1086<br>25 | 0.68045<br><.0001<br>49 |
| **Pyrene** | 0.17193<br>0.3468<br>32 | 0.08797<br>0.5566<br>47 | 0.42260<br>0.0016<br>53 | -0.00233<br>0.9906<br>28 | -0.05721<br>0.7859<br>25 | 0.53600<br><.0001<br>49 |

# Table 2: Correlation (rank) of PAH and adducts: complete case analysis

## The CORR Procedure

| Spearman Correlation Coefficients<br>Prob > \|r\| under H0: Rho=0<br>Number of Observations | | | | |
|---|---|---|---|---|
| | **Benzo_a_anthracene** | **Benzo_a_pyrene** | **Benzo_b_fluoranthene** | **Benzo_ghi_perylene** |
| **Ieno_1_2_3_cd_pyrene** | 0.47840<br>0.0037<br>35 | 0.62087<br>0.0003<br>30 | 0.57383<br>0.0003<br>35 | 0.72480<br><.0001<br>35 |
| **Naphthalene** | 0.39508<br>0.0034<br>53 | 0.44551<br>0.0031<br>42 | -0.02895<br>0.8370<br>53 | 0.41045<br>0.0034<br>49 |
| **Perylene** | 0.59144<br><.0001<br>49 | 0.69353<br><.0001<br>40 | 0.57603<br><.0001<br>49 | 0.74916<br><.0001<br>48 |
| **Phenanthrene** | 0.48846<br>0.0002<br>53 | 0.50308<br>0.0007<br>42 | 0.55138<br><.0001<br>53 | 0.63280<br><.0001<br>49 |
| **Pyrene** | 0.56389<br><.0001<br>53 | 0.61207<br><.0001<br>42 | 0.74884<br><.0001<br>53 | 0.69251<br><.0001<br>49 |

# Table 2: Correlation (rank) of PAH and adducts: complete case analysis

## The CORR Procedure

| Spearman Correlation Coefficients<br>Prob > \|r\| under H0: Rho=0<br>Number of Observations | | | | | | |
|---|---|---|---|---|---|---|
| | **Benzo_k_fluoranthene** | **Chrysene** | **Dibenzo_a_h_anthracene** | **Dibenzofuran** | **Fluoranthene** | **Fluorene** |
| **Ieno_1_2_3_cd_pyrene** | 0.43182<br>0.0096<br>35 | 0.43185<br>0.0096<br>35 | 0.06077<br>0.8437<br>13 | -0.00955<br>0.9586<br>32 | 0.55391<br>0.0006<br>35 | 0.07658<br>0.7947<br>14 |
| **Naphthalene** | 0.08218<br>0.5625<br>52 | 0.45035<br>0.0007<br>53 | 0.20088<br>0.4911<br>14 | 0.17628<br>0.2307<br>48 | 0.26966<br>0.0509<br>53 | 0.27559<br>0.2535<br>19 |
| **Perylene** | 0.69012<br><.0001<br>49 | 0.60201<br><.0001<br>49 | 0.20971<br>0.4718<br>14 | 0.18727<br>0.2235<br>44 | 0.76430<br><.0001<br>49 | 0.02609<br>0.9208<br>17 |
| **Phenanthrene** | 0.44329<br>0.0010<br>52 | 0.47696<br>0.0003<br>53 | 0.02428<br>0.9343<br>14 | 0.36358<br>0.0111<br>48 | 0.73455<br><.0001<br>53 | 0.30912<br>0.1978<br>19 |
| **Pyrene** | 0.68057<br><.0001<br>52 | 0.56698<br><.0001<br>53 | 0.23179<br>0.4252<br>14 | 0.19856<br>0.1761<br>48 | 0.93139<br><.0001<br>53 | 0.17102<br>0.4839<br>19 |

# Table 2: Correlation (rank) of PAH and adducts: complete case analysis

## The CORR Procedure

**Spearman Correlation Coefficients**
**Prob > |r| under H0: Rho=0**
**Number of Observations**

| | Ieno_1_2_3_cd_pyrene | Naphthalene | Perylene | Phenanthrene | Pyrene |
|---|---|---|---|---|---|
| **Ieno_1_2_3_cd_pyrene** | 1.00000<br><br>35 | 0.16584<br>0.3411<br>35 | 0.59175<br>0.0002<br>35 | 0.42649<br>0.0106<br>35 | 0.59192<br>0.0002<br>35 |
| **Naphthalene** | 0.16584<br>0.3411<br>35 | 1.00000<br><br>53 | 0.44992<br>0.0012<br>49 | 0.37948<br>0.0051<br>53 | 0.17542<br>0.2090<br>53 |
| **Perylene** | 0.59175<br>0.0002<br>35 | 0.44992<br>0.0012<br>49 | 1.00000<br><br>49 | 0.74615<br><.0001<br>49 | 0.75818<br><.0001<br>49 |
| **Phenanthrene** | 0.42649<br>0.0106<br>35 | 0.37948<br>0.0051<br>53 | 0.74615<br><.0001<br>49 | 1.00000<br><br>53 | 0.73195<br><.0001<br>53 |
| **Pyrene** | 0.59192<br>0.0002<br>35 | 0.17542<br>0.2090<br>53 | 0.75818<br><.0001<br>49 | 0.73195<br><.0001<br>53 | 1.00000<br><br>53 |

# Principal components analysis of PAH in moss: complete case analsysi of correlation matrix

## The CORR Procedure

| | |
|---|---|
| **3 Variables:** | Dibenzofuran Naphthalene Pyrene |

| Simple Statistics | | | | | | |
|---|---|---|---|---|---|---|
| **Variable** | **N** | **Mean** | **Std Dev** | **Sum** | **Minimum** | **Maximum** |
| **Dibenzofuran** | 48 | 5.97604 | 6.99461 | 286.85000 | 2.37000 | 41.80000 |
| **Naphthalene** | 53 | 13.13594 | 16.30821 | 696.20500 | 3.89000 | 97.70000 |
| **Pyrene** | 53 | 28.51509 | 10.06842 | 1511 | 10.75000 | 68.40000 |

**Pearson Correlation Coefficients**
**Prob > |r| under H0: Rho=0**
**Number of Observations**

| | **Dibenzofuran** | **Naphthalene** | **Pyrene** |
|---|---|---|---|
| **Dibenzofuran** | 1.00000<br><br>48 | 0.96700<br><.0001<br>48 | -0.11355<br>0.4422<br>48 |
| **Naphthalene** | 0.96700<br><.0001<br>48 | 1.00000<br><br>53 | -0.03022<br>0.8299<br>53 |
| **Pyrene** | -0.11355<br>0.4422<br>48 | -0.03022<br>0.8299<br>53 | 1.00000<br><br>53 |

# Principal components analysis of PAH in moss: complete case analsysi of correlation matrix

## The PRINCOMP Procedure

| | |
|---|---|
| **Observations** | 14 |
| **Variables** | 20 |

| Eigenvalues of the Correlation Matrix | | | | |
|---|---|---|---|---|
| | **Eigenvalue** | **Difference** | **Proportion** | **Cumulative** |
| **1** | 7.45922296 | 0.64416189 | 0.3730 | 0.3730 |
| **2** | 6.81506108 | 4.77122931 | 0.3408 | 0.7137 |
| **3** | 2.04383177 | | 0.1022 | 0.8159 |

| Eigenvectors | | | |
|---|---|---|---|
| | **Prin1** | **Prin2** | **Prin3** |
| **_1_Methylnaphthalene** | 0.143863 | 0.307939 | 0.093017 |
| **_2_Methylnaphthalene** | 0.180958 | 0.275066 | -.039925 |
| **Acenaphthene** | 0.183437 | 0.303644 | 0.158278 |
| **Acenaphthylene** | 0.169749 | 0.319706 | 0.175056 |
| **Anthracene** | 0.306689 | 0.185081 | -.009225 |
| **Benzo_a_anthracene** | 0.134408 | -.126970 | 0.334155 |
| **Benzo_a_pyrene** | 0.289231 | -.100647 | 0.048163 |
| **Benzo_b_fluoranthene** | 0.192861 | -.167920 | -.111496 |
| **Benzo_ghi_perylene** | 0.309345 | -.096731 | -.091960 |
| **Benzo_k_fluoranthene** | 0.214107 | -.206196 | -.003249 |
| **Chrysene** | 0.185576 | -.198854 | 0.216759 |
| **Dibenzo_a_h_anthracene** | 0.109765 | -.041866 | 0.717099 |
| **Dibenzofuran** | 0.122313 | 0.335437 | 0.003028 |
| **Fluoranthene** | 0.281742 | -.200033 | -.102066 |
| **Fluorene** | 0.066937 | 0.378875 | -.351013 |
| **Ieno_1_2_3_cd_pyrene** | 0.295171 | -.081074 | -.103359 |
| **Naphthalene** | 0.159224 | 0.312871 | 0.067371 |
| **Perylene** | 0.330341 | -.085464 | -.023130 |
| **Phenanthrene** | 0.268400 | -.082612 | -.250236 |
| **Pyrene** | 0.265774 | -.196370 | -.160832 |

**Principal components analysis of PAH in moss: complete case analsysi of correlation matrix**

**The PRINCOMP Procedure**

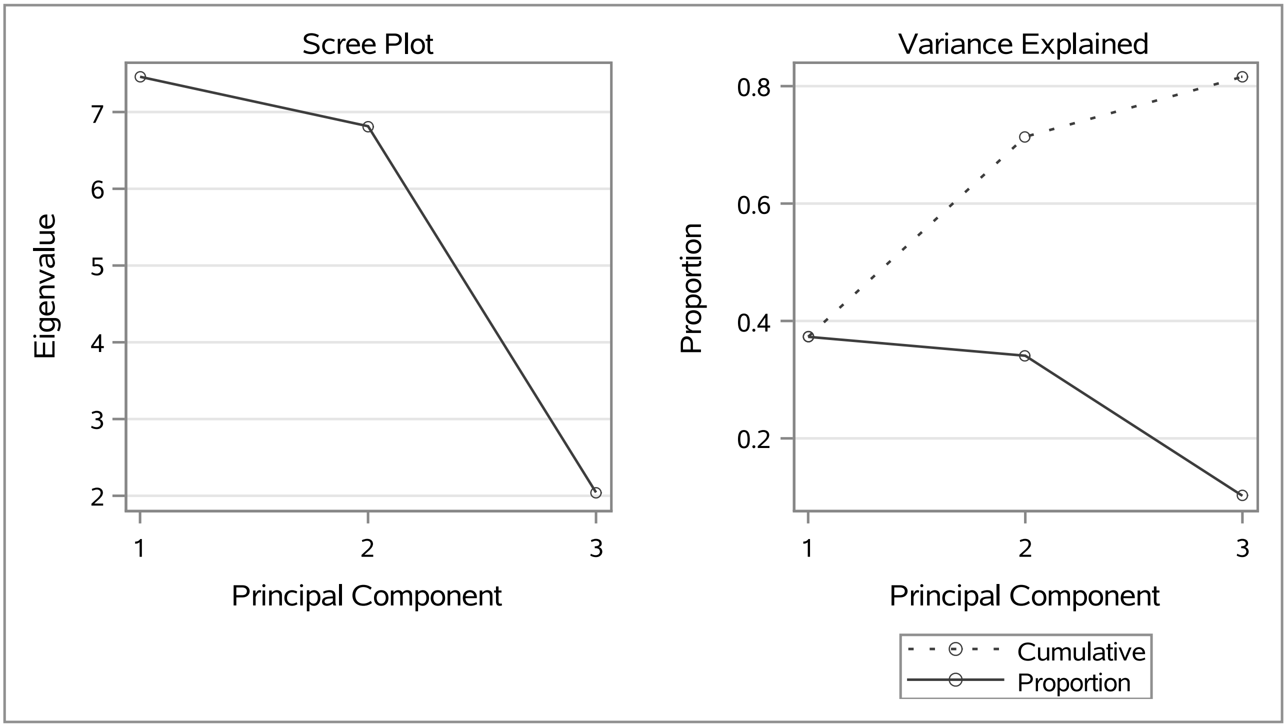

Supplement: S1 File — (PDF) [file pone.0279207.s001.pdf]
