# Supplementary material for: Association of polycyclic aromatic hydrocarbons in moss with blood biomarker among nearby residents in Portland, Oregon
Source: PLoS One. 2022 Dec 19;17(12):e0279207. doi: 10.1371/journal.pone.0279207 (PMC9762581; doi:10.1371/journal.pone.0279207)
Supplement: Attachment — Submitted filename: rebuttal01.docx [file pone.0279207.s002.docx]

Comments of the Editor (ED)

ED2. Please amend your current ethics statement to address the following concerns:

a) Did participants provide their written or verbal informed consent to participate in this study?

b) If consent was verbal, please explain i) why written consent was not obtained, ii) how you documented participant consent, and iii) whether the ethics committees/IRB approved this consent procedure.

RESPONSE: We obtained written consent; study protocol was approved by Drexel University IRB (this was already in the original submission on line 72). The expanded sentence reads, with the new text highlighted: “Drexel University Institutional Review Board approved the study protocol (Protocol number 1402002647), which included written informed consent.”

ED3. Please include your full ethics statement in the ‘Methods’ section of your manuscript file. In your statement, please include the full name of the IRB or ethics committee who approved or waived your study, as well as whether or not you obtained informed written or verbal consent. If consent was waived for your study, please include this information in your statement as well.

RESPONSE: We made the requested change; see response to previous comment.

ED4. In your Data Availability statement, you have not specified where the minimal data set underlying the results described in your manuscript can be found. PLOS defines a study's minimal data set as the underlying data used to reach the conclusions drawn in the manuscript and any additional data required to replicate the reported study findings in their entirety. All PLOS journals require that the minimal data set be made fully available. For more information about our data policy, please see http://journals.plos.org/plosone/s/data-availability.

Upon re-submitting your revised manuscript, please upload your study’s minimal underlying data set as either Supporting Information files or to a stable, public repository and include the relevant URLs, DOIs, or accession numbers within your revised cover letter. For a list of acceptable repositories, please see http://journals.plos.org/plosone/s/data-availability#loc-recommended-repositories. Any potentially identifying patient information must be fully anonymized.

Important: If there are ethical or legal restrictions to sharing your data publicly, please explain these restrictions in detail. Please see our guidelines for more information on what we consider unacceptable restrictions to publicly sharing data: http://journals.plos.org/plosone/s/data-availability#loc-unacceptable-data-access-restrictions. Note that it is not acceptable for the authors to be the sole named individuals responsible for ensuring data access.

We will update your Data Availability statement to reflect the information you provide in your cover letter.

RESPONSE: We are prohibited from sharing data by IRB clearance that governed the project. We do not have permission to share any parts of the data. Copy of IRB approval is attached a material not for external reviewers.

ED5. We note that Figure 1 in your submission contain [map/satellite] images which may be copyrighted. All PLOS content is published under the Creative Commons Attribution License (CC BY 4.0), which means that the manuscript, images, and Supporting Information files will be freely available online, and any third party is permitted to access, download, copy, distribute, and use these materials in any way, even commercially, with proper attribution. For these reasons, we cannot publish previously copyrighted maps or satellite images created using proprietary data, such as Google software (Google Maps, Street View, and Earth). For more information, see our copyright guidelines: http://journals.plos.org/plosone/s/licenses-and-copyright.

 We require you to either (1) present written permission from the copyright holder to publish these figures specifically under the CC BY 4.0 license, or (2) remove the figures from your submission:

   1. You may seek permission from the original copyright holder of Figure 1 to publish the content specifically under the CC BY 4.0 license.

We recommend that you contact the original copyright holder with the Content Permission Form (http://journals.plos.org/plosone/s/file?id=7c09/content-permission-form.pdf) and the following text:

“I request permission for the open-access journal PLOS ONE to publish XXX under the Creative Commons Attribution License (CCAL) CC BY 4.0 (http://creativecommons.org/licenses/by/4.0/). Please be aware that this license allows unrestricted use and distribution, even commercially, by third parties. Please reply and provide explicit written permission to publish XXX under a CC BY license and complete the attached form.”

Please upload the completed Content Permission Form or other proof of granted permissions as an "Other" file with your submission.

In the figure caption of the copyrighted figure, please include the following text: “Reprinted from [ref] under a CC BY license, with permission from [name of publisher], original copyright [original copyright year].”

   2. If you are unable to obtain permission from the original copyright holder to publish these figures under the CC BY 4.0 license or if the copyright holder’s requirements are incompatible with the CC BY 4.0 license, please either i) remove the figure or ii) supply a replacement figure that complies with the CC BY 4.0 license. Please check copyright information on all replacement figures and update the figure caption with source information. If applicable, please specify in the figure caption text when a figure is similar but not identical to the original image and is therefore for illustrative purposes only.

The following resources for replacing copyrighted map figures may be helpful:

 USGS National Map Viewer (public domain): http://viewer.nationalmap.gov/viewer/

The Gateway to Astronaut Photography of Earth (public domain): http://eol.jsc.nasa.gov/sseop/clickmap/

Maps at the CIA (public domain): https://www.cia.gov/library/publications/the-world-factbook/index.html and https://www.cia.gov/library/publications/cia-maps-publications/index.html

NASA Earth Observatory (public domain): http://earthobservatory.nasa.gov/

Landsat: http://landsat.visibleearth.nasa.gov/

USGS EROS (Earth Resources Observatory and Science (EROS) Center) (public domain): http://eros.usgs.gov/#

Natural Earth (public domain): http://www.naturalearthdata.com/

RESPONSE: We removed Figure 1 and any references to it because it is not essential and in fact was not part of the original report of the results. We re-numbered Fig 2 as new Fig 1.

ED6. We note you have included a table to which you do not refer in the text of your manuscript. Please ensure that you refer to Table 1 in your text; if accepted, production will need this reference to link the reader to the Table

RESPONSE: There is only one table in the paper, Table 1, and it is referred as such in text in the original submission. We are unsure where your concern originated from, so please be more specific about what prompted it and we shall find a remedy.

COMMENT Reviewer #1: The possible relationship between atmospheric PAHs and ones in human blood. This is not clear yet, therefore this study did not conclude any relationship between bioindicator and human blood. This result is common and expected. If the authors show evidences for this unclear circumstance, it would be orijinal for the readers.

RESPONSE: We did not study association with atmospheric PAH but rather PAH in moss as indicator of air pollution. It is not obvious to us why atmospheric PAH would not contribute to PAH adducts in blood, given that this is known to occur in highly contaminated settings.

COMMENTS Reviewer#2: (REV2)

REV_01: Although inconclusive, the manuscript entitled "Association of polycyclic aromatic hydrocarbons in moss with blood biomarker among nearby residents in Portland, Oregon," is interesting and may be useful to other researchers. Yet some gaps need to be addressed before publication.

RESPONSE: Thank you for our overall positive assessment. We attempted to address your concerns in the revision as described below in responses to your specific comments.

REV_02: One issue which needs to be addressed stems from surveys of volunteers asking about possible exposure within 48 h hours. This short period is not explained or discussed, although PAHs-adduct half-lives are estimated to be 3-4 months. The authors state that half-lives of PAHs in moss are unknown. However, one would expect, based on PAH stability, they will be at least on a month scale that is much longer than 48h hours.

RESPONSE: This is an excellent point and here we struggled with what can be reliably assessed versus what we wanted to know, reaching a compromise typical of human subjects research such as ours; we added the following explanation: “We recognize that 48 hours is shorter than half-life of PAH but it is a time period that is typically used in epidemiology to more reliably elicit person’s typical behaviors, with a recall on the order of months being unreliable; however, it is customarily assumed that behaviors in the last 48 hours are segregate persons who typical exhibit these behaviors over long periods of time versus those who do not.”

REV_03: It appears the authors omitted the description of sample preparation and analysis of DNA adducts and which specific adducts were analyzed. Alternatively, the reference to the lab performing the work should be provided if this was done as a service.

RESPONSE: We provide only highlights of the method, with reference to exhaustive details already published and included the name of the only lab that performs this adduct assay, as stated “A licensed phlebotomist collected 50 ml of blood by venipuncture. Blood samples were transported by overnighted delivery for quantification of PAH-DNA adduct at the laboratory at Columbia University’s Mailman School of Public Health following protocol of Santella et al. [11-13].”

REV_04: Furthermore, some of the material reported in the results belong to the experimental section. The results section is surprisingly short, extended by the lengthy, simply formatted table. Nevertheless, the authors further narrowed it by focusing on only three representatives PAHs.

RESPONSE: We are unsure which of the results the reviewer wants to see in the description of methods. We aimed to keep the results short and to the point, in keeping with the small nature of the study and a rather straight-forward analysis. We avoided duplication of results in text that are best presented in a tabular format.

REV_05: The major revision of the manuscript needed to address these issues and detailed comments below.
Detailed comments
Line 19. Description of PAHs – which is a full class of compounds as "component" is awkward; please rephrase perhaps as follows "Polycyclic aromatic hydrocarbons (PAHs) are air pollutants.".

RESPONSE: Revised as suggested. Thank you.

REV_06: The direct speech in the abstract using "we" pronoun is refreshing but somewhat overused. To make articles easier to read, some sentences should be rewritten. This is also the case for other sections of the paper.

RESPONSE: We prefer to maintain active voice as it is indeed far easier on the ear than passive voice and it stresses that we, the investigators, did the work, not some abstract entity on our behalf. However, if some specific sentences appear awkward, please identify them and we will gladly attempt to improve the easy of reading.

REV_07: Line 30. It should be "adducts" in the plural.

RESPONSE: Correction made, thank you.

REV_08: Line 76. In this section, the authors carefully describe IRB protocol and data collection. The questionnaire included information of possible exposure to PAHs for 48h. Yet the question is if this is sufficient time for correlations made. The PAHs are quite stable lipophilic compounds that can bioaccumulate. Thus investigation to relationships with longer times seems to be more appropriate. Perhaps authors need to add some explanation for this or can include in the discussion how their approach is appropriate.

RESPONSE: This is a very important issue and we are glad of a chance to explain as detailed above: the 48 hours is a period over which a reliable recall of behaviors can be obtained, deemed in epidemiology to segregate, on average, persons who do or do not exhibit the behavior over longer term.

REV_09: Lines 84, 90. The symbols for degree Celsia are inconsistent; please ensure correct symbols are used everywhere. Usually, "space" is in front of the degree symbol.

RESPONSE: Correction made, thank you.

REV_10: Line 93. Use N2 for nitrogen gas.

RESPONSE: Correction made, thank you.

REV_11: Line 96. "Mass selective detector" does not need to be capitalized. Also, provide mass range and ionization – presumably electron ionization.

RESPONSE: Correction made, thank you. We believe the references to EPA methods and instruments contain all the necessary details to reproduce our work.

REV_12: Line 101. Consider switching the order of 1st two sentences – The current presentation of the 1st sentence without specification of type of statistical analysis is confusing.

RESPONSE: We prefer to retain current order of presentation, because imputation of non-detects does not depend on statistical methods that follow.

REV_13: The experimental section does not address analysis of the PAH-adduct markers.

RESPONSE: We are unsure what the reviewer means because association with the adducts is central to the results section. Please indicate specifically which pieces of information you seek, and we shall try to supply them.

REV_14: Some material from Lines 118-126 including figure 1 belongs to the experimental section.

RESPONSE: Persons you enrolled in the study are the result of application of our method of recruitment. Therefore, these matters are typically presented in the results in our area of scholarship; we appreciate that different conventions exist elsewhere but prefer to structure the narrative in a manner that is most familiar to us. Figure 1 was removed.

REV_15: Lines 128-131 also belong to the experimental section, supporting the study's quality but not being actual results.

RESPONSE: We moved the sentence to methods as suggested.

REV_16: Line 137-139 describes how the study was narrowed, yet correlation for other PAHs could be performed and included in the expanded figure or supplementary materials. Such data would add strength to the paper.

RESPONSE: We added results of principal components analysis as supplementary material, S1 Tables, as suggested.

REV_17: Line 140. The authors mention that specific values for concentration are not informative due to the lack of data on PAHs in moss. It seems it would be useful to compare these results to papers referred in the introduction, justifying the use of this approach.

RESPONSE: We are unsure that comparison to other papers will help in this regard because methods of analysis are far from standardized and there are no criteria values to benchmark any levels in terms of risk.

REV_18: Table 1 need significant revision to achieve a professional look, but also concerning content. PAHs naming nomenclature needs to be revised (there are numerous excellent resources on correct naming). The alphabetic order of PAHs is confusing; it would be better to organize PAH based on volatility or the number of rings and also provide their totals. Correlations with totals of these groupings could be insightful and give a different look at attempted correlation.

RESPONSE: Thank you for prompting us to review the order of PAH in the table. The correction that we made is to move 1-Methylnaphthalene and 2-Methylnaphthalene to appear right above Naphthalene, to restore alphabetical listing. We find alphabetical listing helpful and are unsure what concerns this creates; we are happy to follow specific suggestions, but we followed the spelling and order of PAH from Page 13A-2 of “Compendium of Methods for the Determination of Toxic Organic Compounds in Ambient Air Second Edition Compendium Method TO-13A Determination of Polycyclic Aromatic Hydrocarbons (PAHs) in Ambient Air Using Gas Chromatography/Mass Spectrometry (GC/MS)” published in 1999 by the US EPA (https://www.epa.gov/sites/default/files/2019-11/documents/to-13arr.pdf). Please note that EU regulations also list PAH alphabetically, but we adopted EPA naming convention/spelling (https://www.hbm4eu.eu/hbm4eu-substances/pahs/). Correlation with totals would not be appropriate in this analysis because of problems caused by non-detects (how to add them?) and the fact that there are two principal components (pointing to two independent groupings of PAHs), which makes any calculation of a total (even if possible) to be misleading.
