# Supplementary material for: Association of polycyclic aromatic hydrocarbons in moss with blood biomarker among nearby residents in Portland, Oregon
Source: PLoS One. 2022 Dec 19;17(12):e0279207. doi: 10.1371/journal.pone.0279207 (PMC9762581; doi:10.1371/journal.pone.0279207)
Supplement: Attachment — Submitted filename: rebuttal02.docx [file pone.0279207.s003.docx]

RESPONSE:

We appreciate a chance to consider further revisions and respond to reviewers. We are glad that that there are no specific concerns articulated by the editor and that none of the reviewers appear to identify problems with our methodology. Specific responses to comments and suggestions are listed below, following the reviewers’ comments. We now hope that the manuscript is acceptable for publication but are open to further recommendations for improvement.

Reviewer #1:  PAHs are important air pollutants with very common sources. Many studies are being conducted to establish a relationship with the personal exposures of these pollutants. While it is difficult to establish a statistical relationship between PAHs and human disease even in active sampling and personal exposure studies, it is much more difficult to demonstrate this relationship with bioindicators. (if possible, more discussion is strongly recommended to reveal the relationship).

RESPONSE: We added sentence conveying suggested meaning to the conclusion: “Although we do not challenge the established observation that PAH in environment can cause adverse health effects and produce adducts, our results indicate that it is difficult to demonstrate this relationship with bioindicators in the studied setting. It is plausible that in other environments with a stronger relationship between personal exposure and contamination of properties by PAH bioindicators will prove advantageous over established methods of assessing risk and exposure to humans.”.

Reviewer #2:  The authors addressed the majority of comments, I have just a couple of comments:

I still disagree with naming nomenclature. The EPA is agency providing regulations, limits, and guidelines, not nomenclature. The letters in brackets should be in italics, and the brackets should be square. For details, see NIST reference <https://nvlpubs.nist.gov/nistpubs/Legacy/SP/nistspecialpublication922.pdf>

RESPONSE: We changed brackets to square and the letters in brackets to italics. We trust that the journal will have the final say on the house style.

Line 101.I presume it should be “Restek” column.”

RESPONSE: Correction made; apologies for the typo.

Reviewer #3:  Algae are used to determine PAH levels in outdoor air, but not enough to compare this with human DNA. As you mentioned, it is costly to examine the external environment with classical methods. For this reason, it is being investigated which plant species to use or not to use to determine the atmospheric levels of persistent organic pollutants. The results section is very short.

RESPONSE: We agree with the reviewers views on cost of traditional methods of accessing personal exposure to PAH in general environment and were disappointed that bioindicators were not useful as an alternative in the studied setting. We also agreed that this does not mean that bioindicators cannot be useful in other settings and added two sentences to the conclusions to emphasize this: “Although we do not challenge the established observation that PAH in environment can cause adverse health effects and produce adducts, our results indicate that it is difficult to demonstrate this relationship with bioindicators in the studied setting. It is plausible that in other environments with a stronger relationship between personal exposure and contamination of properties by PAH bioindicators will prove advantageous over established methods of assessing risk and exposure to humans.”. We pride ourselves on construction laconic arguments, hence a short exposition of the results, which speak for themselves through figure and table. Since the reviewer did not specify what they want us to further comment on in the results, we are unable to act on the observation that the section is “very short”.
